# Using Visual Demonstrations of Learning to Promote Age-Integrated Problem Solving

**DOI:** 10.1093/geroni/igab046.1362

**Published:** 2021-12-17

**Authors:** Lisa Borrero

**Affiliations:** University of Indianapolis, Indianapolis, Indiana, United States

## Abstract

Working to dismantle the “othering” of older adults requires active effort to reverse deeply ingrained cultural perceptions and attitudes. As gerontology educators, we are uniquely positioned to “move the needle” on this issue by providing students with the opportunity to engage with older adults in meaningful ways and to see the world from their perspective. In this presentation, visual demonstrations of student learning will be shared that allow students to demonstrate their mastery of course concepts in a creative way and to problem-solve a particular issue by engaging with their own future selves. This approach also allows for a deviation from the routine of written demonstrations of learning by appealing to a different set of student skills. Approaches discussed will include concept maps to deconstruct community collaboration; book bentos to explore optimal aging; multimedia presentations on meaning-making in older adulthood; and a photo elicitation project to address outdoor fall hazards.

